# Special Issue: Virus Receptors and Viral Tropism

**DOI:** 10.3390/v14010068

**Published:** 2021-12-31

**Authors:** Petri Susi

**Affiliations:** Institute of Biomedicine, University of Turku, 20520 Turku, Finland; pesusi@utu.fi

Cell surface receptors play a key role in a virus’ ability to recognize and invade cells and tissues, which basically defines viral pathogenicity. The ongoing COVID-19 pandemic caused by the SARS-CoV-2 virus has shown us the central role of the receptor(s) in the infectious entry of pathogenic viruses. The importance of receptors is further emphasized by their role as vaccine targets. However, as we have been witnessing in the case of SARS-CoV-2, viral surface proteins may rapidly mutate during virus evolution, which may make viruses more infectious and increase the speed by which viruses are able to infect cells. Conversely, viruses may also adapt to avoid neutralizing antibodies, alleviating the effects of vaccines. 

While COVID-19 has severely affected research on topics other than the SARS-CoV-2 virus, this Special Issue, “Virus receptors and viral tropism”, of *Viruses* has managed to attract four original articles and two reviews on a wide range of topics. Papers about RSV receptor interactions, airway epithelial cell tropism, or structural comparisons between the receptor binding of pathogenic viruses are included in the issue and have attracted a lot of interest, with more than 1000 views per article—these numbers are still increasing. The various natures of the papers in addition to the viruses studied indicate that there is still room for receptor studies; we are far from understanding virus receptor–cell surface protein interactions and, more importantly, how they could be used for the benefit of humankind, e.g., in antiviral and vaccine development. I express my sincere thanks to all authors who contributed to this Special Issue, “Virus receptors and viral tropism”.

